# Hydroiodic Acid Additive Enhanced the Performance and Stability of PbS-QDs Solar Cells via Suppressing Hydroxyl Ligand

**DOI:** 10.1007/s40820-020-0372-z

**Published:** 2020-01-24

**Authors:** Xiaokun Yang, Ji Yang, Jahangeer Khan, Hui Deng, Shengjie Yuan, Jian Zhang, Yong Xia, Feng Deng, Xue Zhou, Farooq Umar, Zhixin Jin, Haisheng Song, Chun Cheng, Mohamed Sabry, Jiang Tang

**Affiliations:** 1 Wuhan National Laboratory for Optoelectronics, Huazhong University of Science and Technology, Luoyu Road 1037, Wuhan, 430074 People’s Republic of China; 2 Department of Materials Science and Engineering and Shenzhen Key Laboratory of Nanoimprint Technology, South University of Science and Technology, Shenzhen, 518055 People’s Republic of China; 3 Shenzhen R&D Center of Huazhong University of Science and Technology, Shenzhen, 518000 People’s Republic of China; 4 School of Optical and Electronic Information, Huazhong University of Science and Technology, 1037 Luoyu Road, Wuhan, 430074 People’s Republic of China; 5 National Center for Magnetic Resonance in Wuhan, State Key Laboratory of Magnetic Resonance and Atomic and Molecular Physics, Wuhan Institute of Physics and Mathematics, Chinese Academy of Sciences, Wuhan, 430071 People’s Republic of China; 6 Physics Department, College of Applied Science, Umm Al-Qura University, Mecca, Kingdom of Saudi Arabia; 7 Solar Physics Lab, National Research Institute of Astronomy and Geophysics, Cairo, Egypt

**Keywords:** Hydroxyl ligand, HI additive, Surface passivation, Quantum dots ink, Solar cells

## Abstract

The recent emerging progress of quantum dot ink (QD-ink) has overcome the complexity of multiple-step colloidal QD (CQD) film preparation and pronouncedly promoted the device performance. However, the detrimental hydroxyl (OH) ligands induced from synthesis procedure have not been completely removed. Here, a halide ligand additive strategy was devised to optimize QD-ink process. It simultaneously reduced sub-bandgap states and converted them into iodide-passivated surface, which increase carrier mobility of the QDs films and achieve thicker absorber with improved performances. The corresponding power conversion efficiency of this optimized device reached 10.78%. (The control device was 9.56%.) Therefore, this stratege can support as a candidate strategy to solve the QD original limitation caused by hydroxyl ligands, which is also compatible with other CQD-based optoelectronic devices.

**Electronic supplementary material:**

The online version of this article (10.1007/s40820-020-0372-z) contains supplementary material, which is available to authorized users.

## Highlights

- The hydroiodic acid was explored systematically to modify PbS quantum dots (QDs) ink process, which could remove trap states by hydroxyl ligand and improve iodine passivation on the PbS-QDs surface.
- This strategy solved the essential question of PbS-QDs ink process and showed the favorable application prospects in QDs technology.

**Electronic supplementary material:**

The online version of this article (10.1007/s40820-020-0372-z) contains supplementary material, which is available to authorized users.

## Graphical abstract

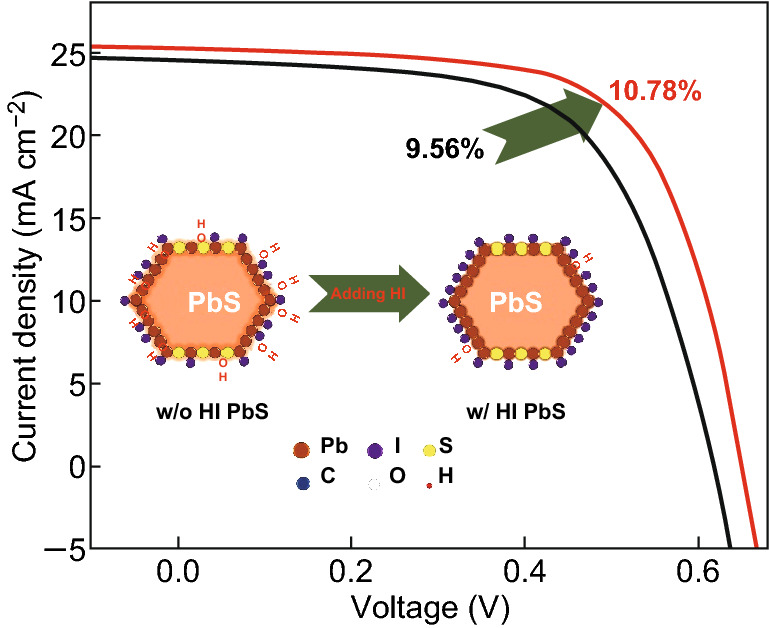

## Introduction

Solution-processed PbS colloidal quantum dots (CQDs) are among the emerging materials for third-generation photovoltaics in view of their simple process [1], large scale [2], low-cost manufacturing [3], and size-dependent bandgaps [4, 5]. In the past decade, surface passivation [6–8] and device architecture [9–14] have been implemented to improve the photovoltaic performances; the efficiencies of PbS QD solar cells have been realized continuous breakthroughs. Recently, QDs-ink process as a new effective technique applied in PbS-QDs solar cells to refresh a new record of power conversion efficiency (PCE) of 12.6% [15]. Therefore, it is a key to further optimize above QDs-ink [15–17] process so as to promote solar cells performance and its industrialization.

**Fig. 1:**
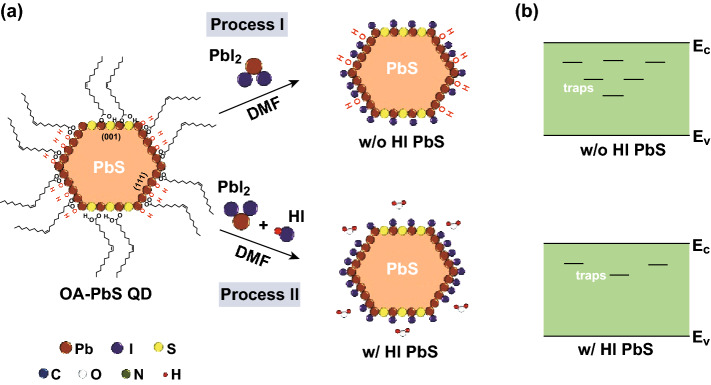
**a** A schematic description of the solution-ligand exchange of PbI_2_ treated PbS-QDs (Process I) and HI-PbI_2_ treated PbS-QDs (Process II). **b** Corresponding trap level evolution of PbS-QDs treated by PbI_2_ and HI-PbI_2_ ligands

In typical CQD synthesis, the QD surface was capped by long-chain oleate surfactants in the representive (001) and (111) facets for passivation and stabilization [6, 18, 19]. Recently, Zherebetskyy et al. [18] reported hydroxyl as a parasitic surface ligand also participated in PbS-QDs surface and played a key role in stabilizing the PbS (111) facet. That is, the synthesized QDs have two well-defined (001) and (111) facets, and the nonpolar (001) facet can be covered by oleate to keep stable state. However, the entire polar (111) facets cannot be fully bonded by the steric hindrance of OA^−^ molecules instead of demanding a smaller hydroxyl ligand (OH) to preserve QDs (Fig. 1a) overall charge neutrality and minimize surface energy [18]. Unfortunately, hydroxyl groups have been proved to introduce sub-bandgap states leading to charge recombination in PbS-QDs solar cells [16, 20, 21], which was detrimental for device performance and photostability. To solve this problem, a series of methods have been developed to eliminate the hydroxyl such as QDs-ink process [16, 17], synthesis precursor selection [22], and thermal annealing [21]. The world record efficiency (12.6%) was achieved from the QD-ink process strategy [15]. However, the hydroxyl as an inherent defect cannot be removed [16, 21] completely in QDs-ink process. Thus, it remains an open challenge for this technology to overcome the hydroxyl effect.

It is well known that the short slab of PbS-QDs for photovoltaic technology is the imperfect surface passivation, which has a remarkable impact on their energy bands [11, 23], trap states [24], carrier diffusion length [8, 13, 25], and stability [3, 21]. We explore a method to remove hydroxyl ligand from PbS-QDs surface and introduce a reliable ligand ion binding to the QD surface for further passivation. Then, we attempt to use one halogenic acid that can be deprotonated to react with hydroxyl ligands and enable halide ions to bind the Pb terminal facet. As the most stable halide ligand for PbS-QDs is iodine ion [2, 11], we perform a suitable amount of mild HI additive in PbI_2_-PbS DMF solution for above motivation.

According to above scenario, here we report a facile HI additive in PbS-QD-ink system to facilitate the control of ligand reactivity and improve the QD-ink stability. It can favor the detachment of hydroxyl groups from (111) facets and promote iodide ligands binding to Pb atoms. The PbS-QDs film treated by the new HI-PbI_2_ ligands has obtained a longer carrier diffusion length and lower trap density. This optimized PbS-QDs solar cells have obtained PCE of 10.78% and exhibited superior operation stability comparing with control devices. The developed ligand additive engineering strategy pronouncedly suppresses the original detrimental effects of hydroxyl and acts as effective strategy to promote the progress of QD technologies [26, 27].

## Materials and Method

### Materials

Zn(CH_3_COO)_2_·2H_2_O (Sinopharm, ≥ 99%), monoethanolamine (Sinopharm, 99%), Ethanedithiol (Sinopharm, ≥ 99%), hydroiodic acid (Sinopharm, 45%), lead oxide PbO (Alfa, 99.9%), oleic acid (OA) (Alfa Aesar, 90%), 1-octadecene (ODE) (Aladdin, ≥ 90%), hexamethyldisilathiane (TMS) (Tci, 95%), octane (Sinopharm, ≥ 95%), acetone (Sinopharm, ≥ 99.5%), ethanol (Sinopharm, ≥ 99.7%), isopropyl alcohol (Sinopharm, 99.7%), 1,2-ethanedithiol (EDT) (Aladdin, 97%), acetonitrile (Sinopharm, ≥ 99.8%), lead iodide (PbI_2_) (Aldrich, 99%), dimethylformamide (DMF) (Aladdin, 99.8%), butylamine (BTA) (Aladdin, 98%), 1-ethyl-3-methylimidazolium iodide (EMII) (Alfa, 97%), tetramethylammonium hydroxide pentahydrate (TMAH) (Aladdin, 97%).

### Fabrication and Characterization

#### Preparation of ZnO Film by Sol–Gel Method

The ZnO precursor was spin-coated on ITO glass at 4000 r min^−1^ for 30 s under ambient environment and then annealed at 320 °C for 12 min.

#### PbS CQDs Synthesis

Oleate-capped PbS CQDs were synthesized under Schlenk-line conditions according to previous reports [2] with slight modifications. A mixture of lead oxide, oleic acid, and 1-octadecenein a flask was degassed and heated for 12 h. After that, TMS was injected into lead oleate solution under vigorous stirring. After purification, the final separated QDs were re-dispersed in octane with a 30 mg mL^−1^ for solar cell fabrication.

#### Device Fabrication

PbS-CQD films fabricated by solution-phase ligand-exchange process serves as the main light-absorbing layer; the oleic acid-capped CQDs (OA-CQDs) could be changed into halide-passivated CQDs under air as described in previous reports [16]. Halide ligand was prepared by PbI_2_-DMF solution for ligand exchange. The OA-CQDs in octane were mixed with the as-prepared DMF solution. The ligand exchanged PbS-CQDs were dried to get CQD powder. The obtained iodide-passivated PbS CQDs were re-dispersed in mixed solvent butylamine (BTA) and *N,N*-dimethylformamide (DMF) with desired concentrations for absorber deposition. For modified QDs-ink process, it is similar to above process and the only difference is the amount of HI additive in PbI_2_ ligand solution. After that, two PbS-EDT layers as an hole extraction layer were fabricated via a layer-by-layer method. Finally, ~ 80 nm Au was deposited by thermal evaporation at low pressure (< 4 × 10^−3^ Pa).

The exact details were shown in supporting information.

## Results and Discussion

A schematic diagram of the solution-phase ligand-exchange process is present in Fig. 1a. Before the ligand exchange, PbS-CQDs are capped with OA^−^ and OH^−^ groups. During the PbI_2_ ligand exchange (Process I), the long-chain and bulky oleate ligands are partially replaced by I^−^ anions, but some hydroxyls hold strong bond with Pb atom of PbS-(111) terminate facet, leading to detrimental effect to device performance [16, 21, 22]. After adding some amount of HI additive in the PbI_2_ ligand exchange (Process II), not only the OA groups are exchanged by iodine ligands, but also the hydroxyls are expected to be eliminated by HI to form a small quantity of free water. For such modified ligand exchange, the trap states are significantly purified comparing with the former one (Fig. 1b). Here, we denoted the HI additive in PbI_2_-PbS-QDs process as w/HI, without HI additive devices as w/o HI or control ones, and oleic acid-capped PbS-QDs as OA-PbS.

To confirm our strategy effect, we measured Fourier-transform infrared (FT-IR) for QD films w/o and w/HI additive to explore the evolution of surface organic groups. The similar weak signal of oleate and hydroxyl ligands is probed in w/o and w/HI-PbS compared to OA-PbS-QDs (Fig. S2a) (C–H_x_ vibrations at 2852–2922 and 1380–1460 cm^−1^, COO^−^ vibrations at 1400–1545 cm^−1,^ OH^−^ vibrations at 3200–3600 cm^−1^) [2, 16], revealing most of OA and hydroxyl ligands were removed by QDs-ink process, only some of residual signal could be detected. For detailed comparison, the OH^−^ vibration [28] peaks of QDs-ink films are highlighted by red square (Fig. S2b). After we added HI in the ligand solution, the OH^−^ peak intensities were reduced two times and the left oleate group signal was also removed. Those results are roughly consistent with our hypothesis, which expect that the HI additive could help to eliminate OH^−^. We further studied the terminal functional groups using ^1^H nuclear magnetic resonance (NMR) spectroscopy. The comparisons among the spectra of background signals (Fig. S3) and ligand exchanged QDs (Fig. S4) indicated that a small amount of oleate residue existed in ink-processed PbS-QDs. After HI was added in PbI_2_-PbS, the hydroxyl peak at 4.48 ppm was suppressed and a broader H_2_O signal at 3.82-4.0 ppm appeared. It further proved that the HI additive could react with OH group by deprotonation reaction to form the free H_2_O.

**Fig. 2:**
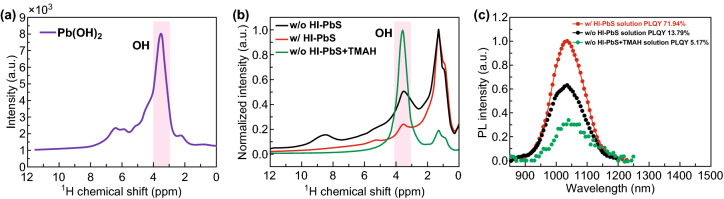
**a** ^1^H NMR for Pb(OH)_2_ powders, **b** ^1^H NMR for w/o, w/HI-PbS, and HI-PbS + TMAH powders. **c** Relative PL intensity of w/o, w/HI-PbS, and HI-PbS + TMAH QDs in solutions. The only H signal for Pb(OH)_2_ was from hydroxyl group, which was located at ~ 3.6 ppm

To enrich the evidence of hydroxyl groups in PbS-QDs, we also employed solid-state ^1^H NMR to detect the hydroxyl from the w/and w/o HI-PbS-QDs powders, which is more close to work condition of these films without any solvents affection. The Pb(OH)_2_ powders were used as reference sample. The w/o HI-PbS + TMAH was obtained by adding 2% mole ratio (related to PbI_2_) TMAH into w/o HI-PbS and was checked by solid-state NMR. As shown in Fig. 2a, b, the w/HI-PbS shows lower intensity of hydroxyl groups than w/o HI-PbS powders. It means that HI additive can suppress hydroxyl in PbS-QDs, which is consistent with IR results. To explore the role of hydroxyl group in these samples, we measured the photoluminescence quantum yield (PLQY) of these powders in solution (Fig. 2c). The PLQY of w/o HI-PbS is 13.79%, w/HI-PbS is 17.94%, while the w/o HI-PbS + TMAH shows lowest PLQY (5.17%), which indicates much hydroxyl along with sub-bandgap states or trap states was suppressed in w/HI-PbS-QDs. Based on above results, we can conclude that the hydroxyls reside in ligand exchanged PbS-QDs ink and introduces trap states in PbS-QDs. Using suitable amount HI as additive in PbS-PbI_2_-DMF system, it can play a role of suppression to the its related trap states.

In order to optimize the content of HI additive in the PbS-QDs, a various amount of HI additive was utilized in PbI_2_ ligand solution. As shown in absorption spectra (Fig. S5a, b), there is no appreciable change in exciton peak between w/o HI and w/HI films with low concentration of HI addition (within 5%). However, more HI additive incorporated in PbI_2_-PbS-QDs solution, the monodispersity of QDs (Fig. S5c, d) degraded because of fusion or decomposition in PbS-QDs. Such results also confirmed by X-ray diffraction and PL spectra (Fig. S6). Therefore, it is necessary using small amount of HI as additive in PbS-QDs ink to preserve the native properties of PbS-QDs without structure degradation.

**Fig. 3:**
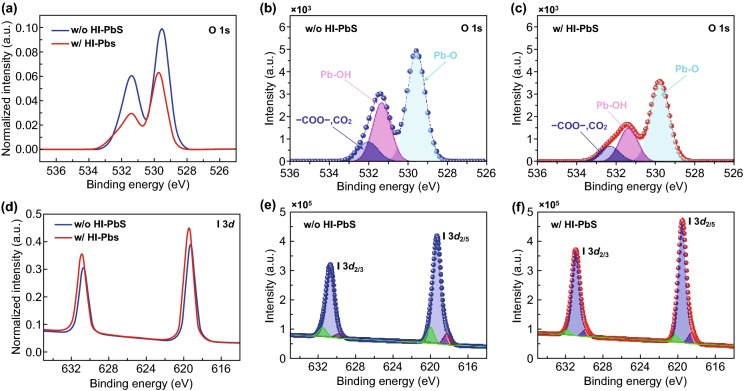
XPS spectra of w/and w/o HI-PbS-QDs. **a** Signal from O 1*s* of two types QDs-ink samples. **b** Signal of O 1*s* from the w/o HI films. **c** Signal of O 1*s* from w/HI films. **d** Signal from I 3*d* of two types QDs-ink samples. **e** Signal of I 3*d* from the w/o HI films. **f** Signal of I 3*d* from w/HI films. The total peak areas of O 1*s* and I 3*d* spectra are normalized to the Pb peak area of the two type samples in **a** and **d,** respectively. To compare the relative values, the real intensity of respective peaks from **b**, **c**, **e,** and **f** are subtracted with background signal

To discuss the changes of the hydroxyl ligands of w/o and w/HI treated PbS-QDs, we performed X-ray photoelectron spectroscopy (XPS) to investigate the species in QD films. As shown in Figs. 3a and S7, the O 1*s* peaks are normalized by Pb peak area, and w/HI films obtain lowest intensity of O 1*s*, revealing the minimum residual OA^−^ ligand compare to others. The O 1*s* peak can be deconvolved into Pb–O (529.6 eV), Pb–OH (531.3 eV), and COO^−^ (532.0 eV) [16, 21]. For OA-PbS film, the O 1*s* peak is dominated by hydroxyl and oleate ligand (Fig.S7b), which are introduced from synthesis process to bind PbS-QDs surfaces [18]. As shown in Fig. 3a–c and Table 1, the Pb-OH peak intensity of w/HI film decreases near two times comparing with w/o HI film, which indicated that hydroxyl had been suppressed by adding HI additive in the ligand solution. It is worth noting that the COO^−^ group has the lowest signal comparing with other two sub-peaks of O 1*s* (Fig. 3b, c); Zherebetskyy et al. attributed this result to the OA^−^ ligands, which has higher possibility for inelastically scattering than OH^−^ ligand, resulting in an underestimate of the actual relative amount of COO^−^ and OH^−^ groups [18]. I 3*d* peaks are also normalized by Pb peak area as shown in Fig. 3d. It is clear that w/HI films hold higher I 3*d* intensity than w/o HI films. From Fig. 3d–f, the Pb-I peak [21, 29, 30] can be deconvolved into three components. From high to low energy, those binding energies for I *3d*_2/5_ are 620.2, 619.5, and 618.4 eV, respectively. (All are constrained to FWHM of < 1.1 eV and within < 0.2 eV deviation.) The higher energy (620.2 eV) component corresponds to iodide ions loosely binding to organic cations or weakly attaching on the QD surface; others lower energy component belongs to iodide binding to Pb atoms [21]. The results of w/HI film show a decrease of the higher energy peaks and a corresponding increase in the lower energy peaks. By calculating the integral area of I 3*d* peak, it can be found the I/Pb ratio increases from 0.65:1 to 0.76:1 (Fig. S8). Based on the O 1*s* and I 3*d* signal changes in w/o and w/HI films, we consider that this effect is attributed to the reduced OH groups and an increase in iodide atoms that are strongly bound to the surface of the QDs. In view of the above results (FT-IR, NMR, and XPS), we can conclude that HI acting as additive reacts with hydroxyl ligand via a deprotonation reaction and enables iodide to bind on PbS-QDs surface with enhanced passivation. It should be noted a similar work [31] has implemented in big size QDs-based infrared solar cells. They focused on the removal of original ligand and improvement in CQD packing; there was limited elaboration for the content changes and evolution between hydroxyl and iodine. Our work paid more attention to the suppression of hydroxyl and passivation of iodine ligand, therefore, made this evolution process more clear.

**Table 1:** Fitting parameters and quantitative analysis of O 1*s* spectra of w/o and w/HI PbS-QDs films in Fig. 3

PbS-QDs	Component	Peak	FWHM	Area (%)	Component ratio
w/o HI PbS-QDs	Pb–O	529.6	1.1	55.7	0.12
	**Pb**–**OH**	531.3	1.2	33.6	**0.071**
	COO,CO_2_	532.0	1.2	10.7	0.022
w/HI PbS-QDs	Pb–O	529.7	1.1	59.7	0.084
	**Pb**–**OH**	531.4	1.2	28.0	**0.032**
	COO,CO_2_	532.3	1.2	12.3	0.017

The bold words were marked to highlight the changes of trap groups, OH group in QDs, in the XPS results

**Fig. 4:**
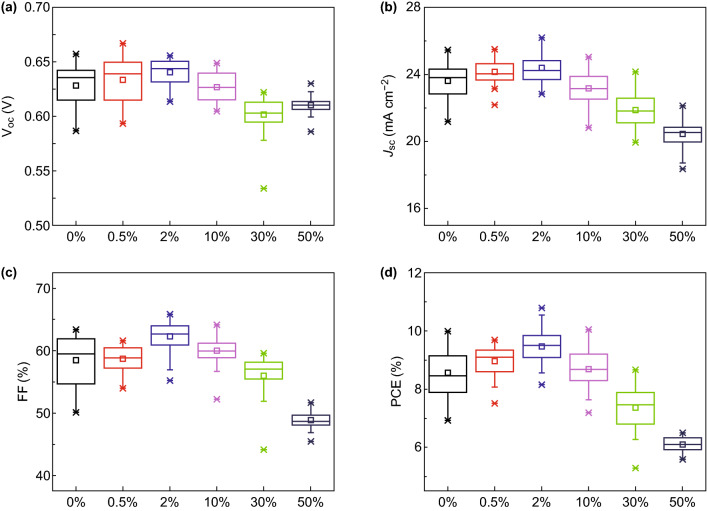
Device performance parameters of **a** *V*_oc_, **b** *J*_sc_, **c** FF, and **d** PCE distribution box with different mole ratio of HI acid added in PbI_2_-PbS solution

To identify the effects of HI additive, we systematically optimized the amount of HI additive in PbI_2_-DMF ligand solution (Fig. 4) and the thickness of absorber for both w/and w/o HI devices (Fig. S9). In w/HI devices, the optimized mole ratio to PbI_2_ is 2%, which achieve the maximum efficiency (10.78%) and the maximum *J*_sc_ (27.86 mA cm^−2^) (average *J*_sc_ is ~ 25.88 mA cm^−2^) with the absorber thickness of ~ 420 nm. With the same thickness, the w/o HI device only obtain maximum *J*_sc_ of 23.65 mA cm^−2^ and average *J*_sc_ of 22.74 mA cm^−2^, indicating the carriers diffusion length of w/o HI devices is shorter than w/HI ones.

**Fig. 5:**
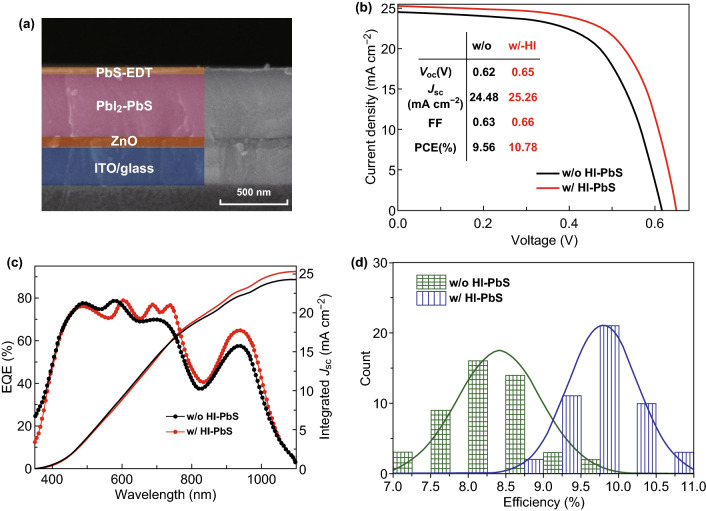
Device architecture and performances. **a** The PbS-QDs solar cell device structure. **b** Representative *J*–*V* characteristics of w/o and w/HI devices under simulated AM 1.5G illumination (100 mW cm^−2^). **c** EQE spectra and integrated *J*_sc_ of w/and w/o HI devices. **d** Statistic histograms of PCE for above two kinds of devices

The current density–voltage (*J*–*V*) characteristics of optimal w/HI and w/o HI devices are shown in Fig. 5b and the device parameters are summarized in inset table. The optimal w/HI-PbS solar cell reaches a PCE of 10.78% (average value ~ 10.37%) with a *V*_oc_ of 0.65 V, a *J*_sc_ of 25.26 mA cm^−2^, and an FF of 0.66, which is superior to the w/o-based device with an optimal PCE of 9.56% (*V*_oc_ = 0.62 V, *J*_sc_ = 24.48 mA cm^−2^, and FF = 0.63). The EQE of both devices are shown in Fig. 5c. At the 930–940 nm exciton peaks, w/HI devices reach 65%. On contrary, the control device value is just close to 53%. We consider this improvement related to suppression of the hydroxide species and enhancement of iodide passivation for w/HI films. The w/HI device shows narrower efficiency distribution, and their average efficiency is much higher than control devices. The statistic histograms of PCE for w/and w/o HI treated PbS-QDs solar cells are summarized in Fig. 5d. Device physical characterizations were further utilized to unfold the background mechanism.

**Fig. 6:**
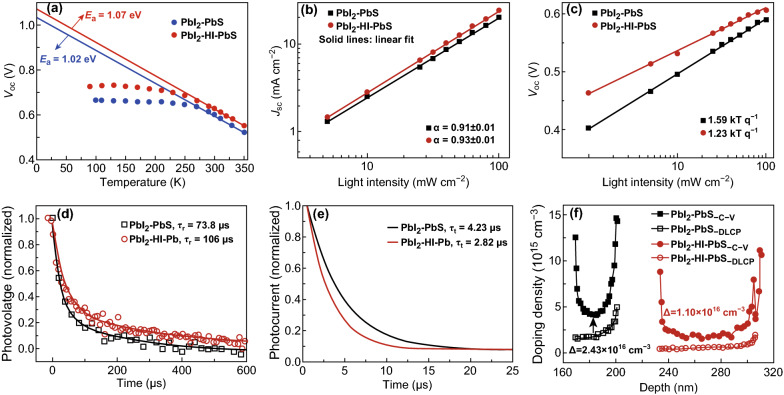
Physical characterizations of w/o and w/HI devices. **a** Temperature dependence of *V*_oc_ under 100 mW cm^−2^ light intensity. Solid line: linear fits. **b** Light-intensity dependence of *J*_sc_. Solid lines: linear fits. **c** Light-intensity dependence of *V*_oc_. Solid lines: logarithmic fits. **d** Charge recombination and **e** transport lifetime calculated by TPV and TPC, respectively. **f** Interfacial defects analysis from *C*–*V* and DLCP results

According to the *J*–*V* curve, the performance enhancement of w/HI PbS-QDs-based devices mainly came from the improvement in *V*_oc_ and FF. In order to gain insight into the physical origins of the improvement performance, we firstly measured the temperature-dependent *J*–*V* characteristics for generation-recombination in these devices. By extrapolating the *V*_oc_ to *T* = 0 K (Fig. 6a), the activation energy [32, 33] of w/and w/o HI devices are 1.07 and 1.02 eV, respectively. Both of them are less than the bandgap of PbS-QDs (*E*_g_ ~ 1.36 eV), indicating obvious existence of interfacial recombination in these devices. And the activation energy value of w/HI device is slightly higher than that of the w/o HI devices, which is ascribed to the better passivation in the interface of ZnO and QDs. To investigate the recombination mechanisms of above two kinds of devices, the light-intensity dependences of *J*_sc_ and *V*_oc_ were measured to get the device ideality factor n [9, 32]. As shown in Fig. 6b, c, the slope of the plot for w/HI device is much lower (1.23 kT/q) than that of control device (1.59 kT/q), which indicate w/HI devices greatly reduced the trap-assisted recombination [22, 32]. Hence, it can help to improve *V*_oc_ and FF.

We further characterized charge-transfer and recombination kinetics in above two kinds of solar cells. We used transient photovoltage decay under the open-circuit condition to characterize solar cells. The extracted charge-recombination lifetime (*τ*_r_) of w/HI devices is substantially longer than that of control device (106 versus 73.8 μs) (Fig. 6d). In Fig. 6e, the obtained charge-transport time of w/HI devices (*τ*_t_ ~ 2.82 μs) is shorter than the value of control one (*τ*_t_ ~ 4.23 μs), indicating the faster photocarrier transport in w/HI devices. From *V*_oc_ and *J*_sc_ transient decay dynamics, it concluded that both interfacial and bulk qualities of w/HI devices were improved compared with control devices.

Based on above analyses, it demonstrated that HI treated PbS-QDs suppressed the defects at ZnO/PbS-QDs heterojunction interface and QDs bulk layers. Capacitance–voltage (*C*–*V*) profiling and deep-level capacitance profiling (DLCP) measurements were carried out on these devices to investigate the related defect information. In general, *C*–*V* content includes responses from free carriers [34], defects in the bulk, and interfacial defects, while DLCP result is only sensitive to free carriers and bulk defects [33, 35]. Thus, the difference between *N*_*C*–*V*_ (defect density calculated from *C*–*V* measurement) and *N*_DLCP_ (defect density calculated from DLCP measurement) reflects defect density [33] at the ZnO/PbS interface. As shown in Fig. 6f, the above two devices based DLCP curves are not overlapped and the value of w/HI device is lower than w/o HI one. Combining *C*–*V* and DLCP measurements, we calculated the interfacial defect density as 4.07 × 10^10^ cm^−2^ in w/HI treated one, which was near three times lower than that in w/o HI treated device (1.35 × 10^11^ cm^−2^). In order to obtain the QD surface defect density of bulk PbS films, we carried out the DLCP measurement at low (150 K) and room temperature (300 K) (Fig. S10); the difference of doping density is bulk trap states [35, 36]. The extracted surface defect concentration of bulk PbS films from w/HI device is ~ 1.5 × 10^15^ cm^−3^, which is threefold lower than w/o HI PbS-QDs device (~ 5×10^15^ cm^−3^). These results are in accordance with the results of TPV (Fig. 6d) and SCLC analysis (Fig. S11).

**Fig. 7:**
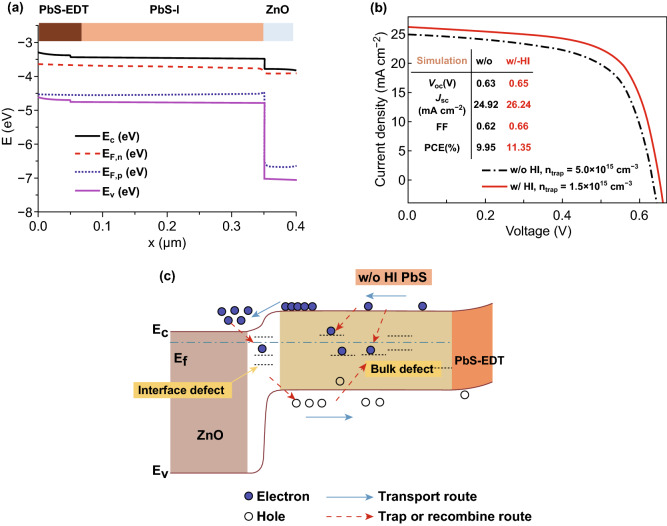
**a** The energy band from SCAPS modeling for PbS-QDs solar cell. **b** Current–voltage characteristics under AM 1.5G illumination for w/and w/o HI devices. **c** Schematic illustration of proposed photocarrier transport and transfer for w/o and w/HI devices based on simulation and device physics results

To confirm the above physical results and deduce the energy band structure, we built a one-dimension model of optoelectronic device (Fig. 7a) that took into account the electron affinity of CQDs with w/and w/o HI treatments, absorption profiles, trap density, and carrier mobility referring from our previously work [37, 38] or reported values from iodide-passivated films [8] (Fig. S12 and Table S2). From these simulation results (Fig. 7b), here is showed similar results with real w/o and w/HI devices. That is, the lower trap density of device would get the better performance. From the simulation results, HI additive could enhance the performances (*V*_oc_, *J*_sc_, and FF) for PbS-QDs devices by suppressing the detrimental effect of hydroxyl ligands. Based on the above conclusions, a schematic illustration of proposed photocarrier transport and transfer for w/o and w/HI devices were plotted as shown in Fig. 7c. For w/o HI-based devices, the sub-bandgap states induced by hydroxyl ligand would trap the carriers and promote non-radiative recombination, leading to lower *J*_sc_ and *V*_oc_. Those results confirmed the importance of removing hydroxyl and its detrimental effect in PbS-QDs for achieving high efficiency photovoltaics.

As HI-processed PbI_2_-PbS solar cells suppressed hydroxyls and converted them with iodine passivation, which was expected to hold better stability. Thus, we measured the device ambient storing stability at room temperature. As shown in Fig. S13a, w/HI devices could keep ~ 60% of their original PCE after 1500 min under illumination, which were much better than w/o devices (~ 40% after 1500 min). The main performance decays were from the loss of *J*_sc_ and FF (Fig. S13b-d), which may be degraded by oxidation effects [2, 11]. For higher HI concentration (50%)-processed-PbS solar cells, they decayed more seriously to 50% of their initial PCE only after 60 min illumination. This fast degradation was mainly caused by overtreatment by HI, leading to the fusion or etching in PbS-QDs, which led to be more sensitive to ambient atmosphere. It also indicated that the amount of additive HI needs to be precise controlled.

Compared with previous work by Jo et al. [31], the motivation and key finding of them are different to ours. They mostly focused on large-diameter (4–6 nm, *E*_g_ < 1.1 eV) CQDs for harvest energy in the infrared region of the solar spectrum. They employed the hydroiodic acid in order to increase the chemical reactivity to facilitate high CQD packing and passivation for large size infrared CQDs (IR-QDs). Our work focused on the improved performance for wider-bandgap CQDs whose bandgaps were within the range of 1.3–1.5 eV, closed to the idea band gap for single junction solar cell. In our case, it is not the key problem to remove the oleate ligand after solution-processed ligand-exchange method referring from our FT-IR spectra. And the wider-bandgap CQDs of ours used for single junction solar cells focused on the elimination for sub-bandgap states and improvement in passivation. Therefore, we paid more attention about device physics characterization to know about the mechanism of hydroxyl ligand to PbS-QDs solar cell performance. Meanwhile, we extracted the optimal recipe for hydroxyl removal treatment. In general, the reported work [31] and ours focused on different motivations and different size QD for harvesting different region of the solar spectrum. And these two works can hold the complementary role for each other.

## Conclusions

In summary, we demonstrated a halide ligand additive strategy to suppress hydroxyl ligands and convert them with iodine ligand passivation. The approach allowed the halide acid, HI, to access target Pb-OH bonding based on a deprotonation reaction between HI additive and hydroxyl ligands. Utilizing optimal concentration HI treatment, QDs could obtain higher I/Pb ratio (0.75:1) with improved passivation on QDs surface. Such treatment strategy promised thicker absorber layer with higher mobility of carriers in solar cells, delivering a PCE of 10.78%. Systematic physical analyses to the improvement mechanism were originated from the suppression of interfacial and bulk defect density. Thus, the device *V*_oc_ and FF could be significantly improved as well as the device stability. The present ingenious strategy was expected to continuously promote PbS-QDs ink technology, and also be applicable for other CQD-based optoelectronic devices.

## Electronic Supplementary Material

Below is the link to the electronic supplementary material.

Supplementary material 1 (PDF 1519 kb)
