# Supplementary material for: Hydroiodic Acid Additive Enhanced the Performance and Stability of PbS-QDs Solar Cells via Suppressing Hydroxyl Ligand
Source: Nanomicro Lett. 2020 Jan 24;12:37. doi: 10.1007/s40820-020-0372-z (PMC7770827; doi:10.1007/s40820-020-0372-z)
Supplement: Supplementary file 1 — Supplementary material 1 (PDF 1519 kb) [file 40820_2020_372_MOESM1_ESM.pdf]

Supporting Information for

Hydroiodic Acid Additive Enhanced the Performance and Stability of PbS-QDs Solar Cells via Suppressing Hydroxyl Ligand

Xiaokun Yang[1, 2, 3, +], Ji Yang[1, +], Jahangeer Khan[1], Hui Deng[1, 3], Shengjie Yuan[1, 3], Jian Zhang[1], Yong Xia[4], Feng Deng[5], Xue Zhou[5], Farooq Umar[1], Zhixin Jin[1, 2], Haisheng Song[1, 3, *], Chun Cheng[2, *], Mohamed Sabry[6, 7], Jiang Tang[1, 3]

[1]Wuhan National Laboratory for Optoelectronics, Huazhong University of Science and Technology, Luoyu Road 1037, Wuhan 430074, People's Republic of China

[2]Department of Materials Science and Engineering and Shenzhen Key Laboratory of Nanoimprint Technology, South University of Science and Technology, Shenzhen 518055, People's Republic of China

[3]Shenzhen R&D Center of Huazhong University of Science and Technology, Shenzhen 518000, People's Republic of China

[4]School of Optical and Electronic Information, Huazhong University of Science and Technology, 1037 Luoyu Road, Wuhan 430074, People's Republic of China

[5]National Center for Magnetic Resonance in Wuhan, State Key Laboratory of Magnetic Resonance and Atomic and Molecular Physics, Wuhan Institute of Physics and Mathematics, Chinese Academy of Sciences, Wuhan 430071, People's Republic of China

[6]Physics Department, College of Applied Science, Umm Al-Qura University, Kingdom of Saudi Arabia

[7]Solar Physics Lab, National Research Institute of Astronomy and Geophysics, Egypt

[+]Xiaokun Yang and Ji Yang contributed equally to this work

*Corresponding authors. E-mail: songhs-wnlo@mail.hust.edu.cn (H. Song), chengc@sustc.edu.cn (C. Cheng)

S1 Experimental Section

S1.1 Materials

$Zn(CH_3COO)_2·2H_2O$ (Sinopharm, ≥99%), Monoethanolamine (Sinopharm, 99%), Ethanedithiol (Sinopharm, ≥99%), Hydroiodic acid (Sinopharm, 45%), Lead Oxide PbO (Alfa, 99.9%), Oleic Acid (OA) (Alfa Aesar, 90%), 1-octadecene (ODE) (Aladdin, ≥90%), Hexamethyldisilathiane (TMS) (Tci, 95%), Octane (Sinopharm, ≥95%), Acetone (Sinopharm，≥99.5%), Ethanol (Sinopharm, ≥99.7%), Isopropyl alcohol (Sinopharm, 99.7%), 1,2-ethanedithiol (EDT) (Aladdin, 97%), Acetonitrile (Sinopharm, ≥99.8%), Lead iodide (PbI_2) (Aldrich, 99%), Dimethylformamide (DMF) (Aladdin, 99.8%), Butylamine (BTA) (Aladdin, 98%), 1-ethyl-3-methylimidazolium iodide (EMII) (Alfa, 97%), Tetramethylammonium hydroxide pentahydrate (TMAH) (Aladdin, 97%).

S1.2 Fabrication and Characterization

S1.2.1 Preparation of ZnO Film by Sol-gel Method

The ZnO sol-gel precursor was prepared according to a previously reported procedure [S1]. The ZnO precursor was spin-coated on ITO glass at 4000 r min^{-1} for 30 s under ambient environment, followed by annealing 320 °C for 12 min. This process was repeated a few times to reach the required thickness.

S1.2.2 Lead Sulfide Colloidal Quantum Dots (PbS CQDs) Synthesis

Oleate-capped PbS CQDs were synthesized under Schlenk-line conditions according to previous reports [S2] with slight modifications [S1]. A mixture of lead oxide (4 mmol, 0.9 g), oleic acid (9.5 mmol, 3 mL) and 1-octadecene (20 mL) in a flask was gradually increased to target temperature and degassed for 12 h. After that, 2 mmol TMS was dissolved in 20 mL ODE, and then injected quickly into lead oleate solution under vigorous stirring at 120 °C. The reaction took a few minutes and then it cooled to room temperature naturally. QDs were purified in air by adding octane to dissolve and precipitated by acetone with centrifugation. This process was repeated three times, and the final separated QDs were re-dispersed in Octane with a 30 mg mL^{-1} for solar cell fabrication.

S1.2.3 Device Fabrication

PbS CQD films fabricated by solution-phase ligand-exchange process serves as the main light-absorbing layer, the oleic acid-capped CQDs (OA-CQDs) could be changed into halide-passivated CQDs under air as described in previous reports [S3]. Halide ligand was prepared by PbI_2-DMF solution (691 mg/5 mL) for ligand exchange. 5 mL of OA-CQDs in octane (15 mg mL^{-1}) were mixed with the as-prepared DMF solution. For complete transfer of CQD toward DMF, this mixed solution was vortexed for 1.5 min at room temperature. The ligand-exchanged solution, CQD dispersed in DMF was washed by octane three times for removing remained residues. After washing, toluene was added to the ligand exchanged solution for precipitation of CQD, and the CQDs were totally collected by centrifugation. The

PbS CQDs were dried in vacuum for 20 min to get CQD powder. The obtained iodide-passivated PbS CQDs were re-dispersed in mixed solvent Butylamine (BTA) and *N*,*N*-dimethylformamide (DMF) with desired concentrations for absorber deposition. The volume ratio of this mixed solvent is V_{BTA}: V_{DMF} = 9:1. For modified QDs-ink process, it is similar to above process and the only difference is the amount of HI additive in PbI_2 ligand solution. We adding HI (45%) with different mole ratio related to PbI_2 in PbI_2-DMF solution. For example, the 2% HI need add 3 μL HI solution into 5 mL PbI_2-DMF solution. After that, two PbS-EDT layers as an hole extraction layer were fabricated via a layer-by-layer method. OA-CQDs were dropped onto pre-deposited PbS-PbI_2 layer and spin-cast at 2500 rpm for 18 s, and then a 0.01 vol% 1,2-Ethanedithiol in Acetonitrile solution was loaded on the film for 30 s, then spin-casted at 2500 rpm for 10s, followed by washing with acetonitrile for 2 times. This process repeated one time more. Finally, ~80 nm Au was deposited by thermal evaporation at low pressure (< 4 × 10^{-3} Pa).

The w/o HI-PbS+TMAH powders or films were similar to above modified QDs-ink process, the only difference is the HI additive are replaced by TMAH (with 2% mole ratio related to PbI_2).

For EMII and TMAH treated device, the EMII-methanol solution (10mg/ml) or TMAH-methanol solution (10 mg mL^{-1}) was dropped on QD surface, kept for 1 min and 20 s respectively, and then span for 20 s. The EMII (TMAH) ligand exchanged film was washed three times by methanol to remove the non-reacted ligand residue. The above EMII (TMAH) treated PbS QD was repeated for 8-10 times to reach the required absorber thickness. At the end of active layer, another two layers of PbS-EDT QDs were spin-coated similar to above process.

S1.2.4 Device Characterization

The current density-voltage (*J-V*) characteristics were measured using Keithley 2400 (*J-V*) digital source meter under simulated AM1.5G (100 mW cm^{-2}) illumination from a 450 W Xenon lamp (Oriel, Model 9119, Newport) as the light source in air at room temperature. The light intensity was calibrated with a standard Si solar cell (Oriel, Model 91150V, Newport). The device was covered with a metal mask with an aperture area of 0.09 cm [S2] during efficiency measurement. The external quantum efficiency (EQE) measurements were taken by a home-made setup containing a Keithley 2400 Source Measure unit and Newport monochromator.

S1.2.5 Characterization of Material and Device

The absorbance of PbS CQDs was obtained using UV-visible spectroscopy (PerkinElmer instrument, Lambda 950). The mobility of the CQD films was measured using space-charge-limited current (SCLC) model [S4] with an electron-only or a hole-only device of ITO/CQDs/Al and ITO/CQDs/Au respectively. The dark *J-V*

curve wasrecorded using a B1500A semiconductor characterization system and used to calculate the mobility of carriers by Eq. S1:

$$J_D = \frac{9\varepsilon_0\varepsilon\mu V_b^2}{8L^3} \quad \text{(S1)}$$

where L is the thickness of the single carrier device, ε_0 is the vacuum permittivity, ε is the relative dielectric constant for the PbS-CQDs, which is obtained from *C-f* measurements (Fig. S16), the trap-state density can be extracted by form Eq. S2 [S5]

$$V_{\text{TFL}} = \frac{e n_{\text{trap}} L^2}{2\varepsilon_0\varepsilon} \quad \text{(S2)}$$

where n_{trap} is the trap density of the PbS-QDs layer, The V_{TFL} and V_{b} is extracted from Fig. S13.

X-ray photoelectron spectroscopy (XPS) and Ultraviolet photoelectron spectroscopy (UPS) measurements were carried out in a Kratos AXIS Ultra-DLD with an Al Kα radiation source. Scanning electron microscopy (SEM) images were obtained using FEI Nova Nano SEM 450. Photoluminescence (PL) measurements were carried out using home-made system with 800 nm laser (Ti-Sapphire laser) from School of optical and electronic information. FT-IR spectra were obtained by performing FT-IR spectrometer (Bruker Vertex 70), the transmission mode FT−IR samples were taken from PbS-QDs powders mixed with potassium bromide. All the solution-state ^{1}H NMR experiments were carried out on a Bruker Avance III 600 instrument with scanned 128 times, all the Solid-state ^{1}H NMR were recorded on a Bruker Avance III 400M spectrometer with scanned 156 times. All of solid NMR samples were dewatered several hours under vacuum and heated.

The capacitance–voltage (C_p – V) measurements were acquired with an Agilent 4200A at a frequency of 10 kHz and AC signal of 50 mV, scanning from -1 to +1 V, with a step size of 50 mV. The built-in potentials (V_{bi}) of the devices were obtained from a Mott-Schottky plot. The depleted width of QD layer (W_{D}) at zero bias was accorded to Eqs. S3 and S4:

$$W_D = \frac{1}{N_A}\left[\frac{2\varepsilon_{QD}\varepsilon_0}{q\left(\frac{1}{N_D}+\frac{1}{N_A}\right)}V_{bi}\right]^{\frac{1}{2}} \quad \text{(S3)}$$

and

$$N_A = \frac{2}{A^2 q\varepsilon_{QD}\varepsilon_0\frac{d}{dV}(\frac{1}{C^2})} \quad \text{(S4)}$$

where N_A and ε_{QD} were the PbS CQD carrier density and dielectric constant respectively, ε_{QD} was taken from *c-f* measurement (Fig. S16) like previous work [S1, S6], ε_0 is the vacuum permittivity, N_D is the ZnO carrier density from the Hall Effect results, and V_{bi} is the built in potential of QDs devices.

The transient photovoltage (TPV) and **transient photocurrent decay (TPC)** measurements were carried out using home-made system in dark condition. A ring of red light-emitting pulse diode (LED, Lumiled) controlled by a fast solid-state switch (the pulse widths were 1 ms). The transient photocurrent was measured using 40 ohm external series resistance to operate the device in short-circuit condition. Similarly, transient photovoltage was applied using 1 M ohm external series resistance to operate the device in open-circuit condition. The voltage output was recorded using an oscilloscope directly with connecting the measured solar cells. The TPC and TPV results were fitted to a mono-exponential decay function to extract the transport and recombination time [S7], respectively. The charge transport (τ_t) and recombination time (τ_r) were defined as the time interval during which the photocurrent or photovoltage decays to 1/e of their initial value immediately after excitation.

Drive-level capacitance profiling (DLCP) measurement of the devices (ITO/ZnO/CQD/Au) was performed with variant amplitude (14-140 mV) and frequency (100–100 kHz) [S3]. Capacitance with respect to amplitude was obtained to correct the capacitance value at higher orders, following Eq. S5:

$$C = C_0 + C_1 dV + C_2 (dV)^2 + \cdots \quad \text{(S5)}$$

Density of state for both the w/o and w/ HI PbS-QDs with the variation in frequency were calculated using Eq. S6 [S8]:

$$N = \frac{C_0^3}{2q\varepsilon A^2 C_1} \quad \text{(S6)}$$

Solar cell device Simulations were performed using SCAPS software [S9] to ensure the energy band gap schematic and accept the guidance for device performance improvement. The complete set of simulation parameters in this simulation based on previous work [S3, S10] with slightly modification [S6], as shown in Fig. S12 and Table S2.

S2 Supplementary Figures and Tables

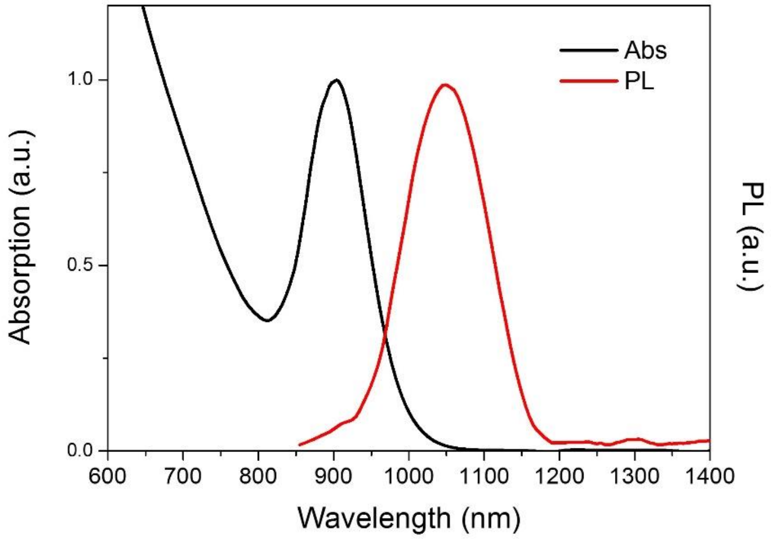

Fig. S1 Absorption and photoluminescence spectra of PbS-OA QDs dispersed in octane solution

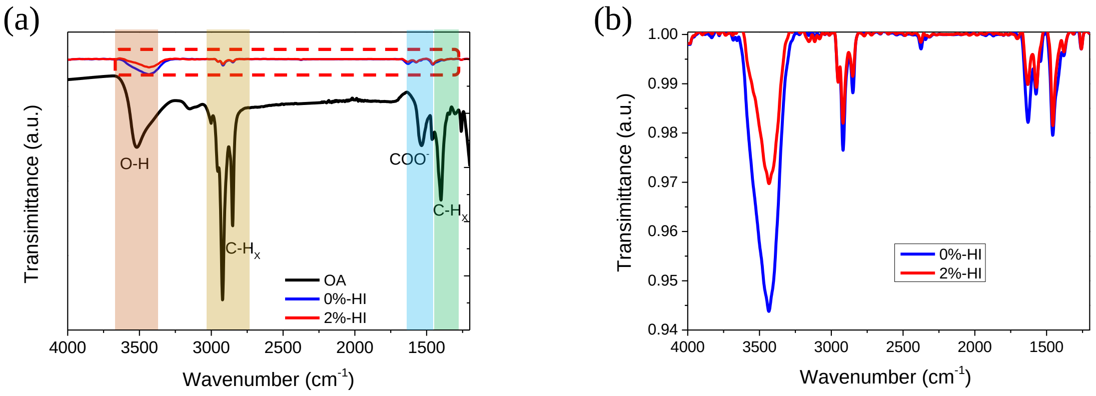

Fig. S2 a Fourier transform infrared (FT-IR) spectra of PbS QDs capped by OA, w/o and w/ HI-PbI_2 mixed ligands. **b** The dashed square region was zoom in to show the residual hydroxyl and oleate ligand. All of these samples were prepared into powders to check

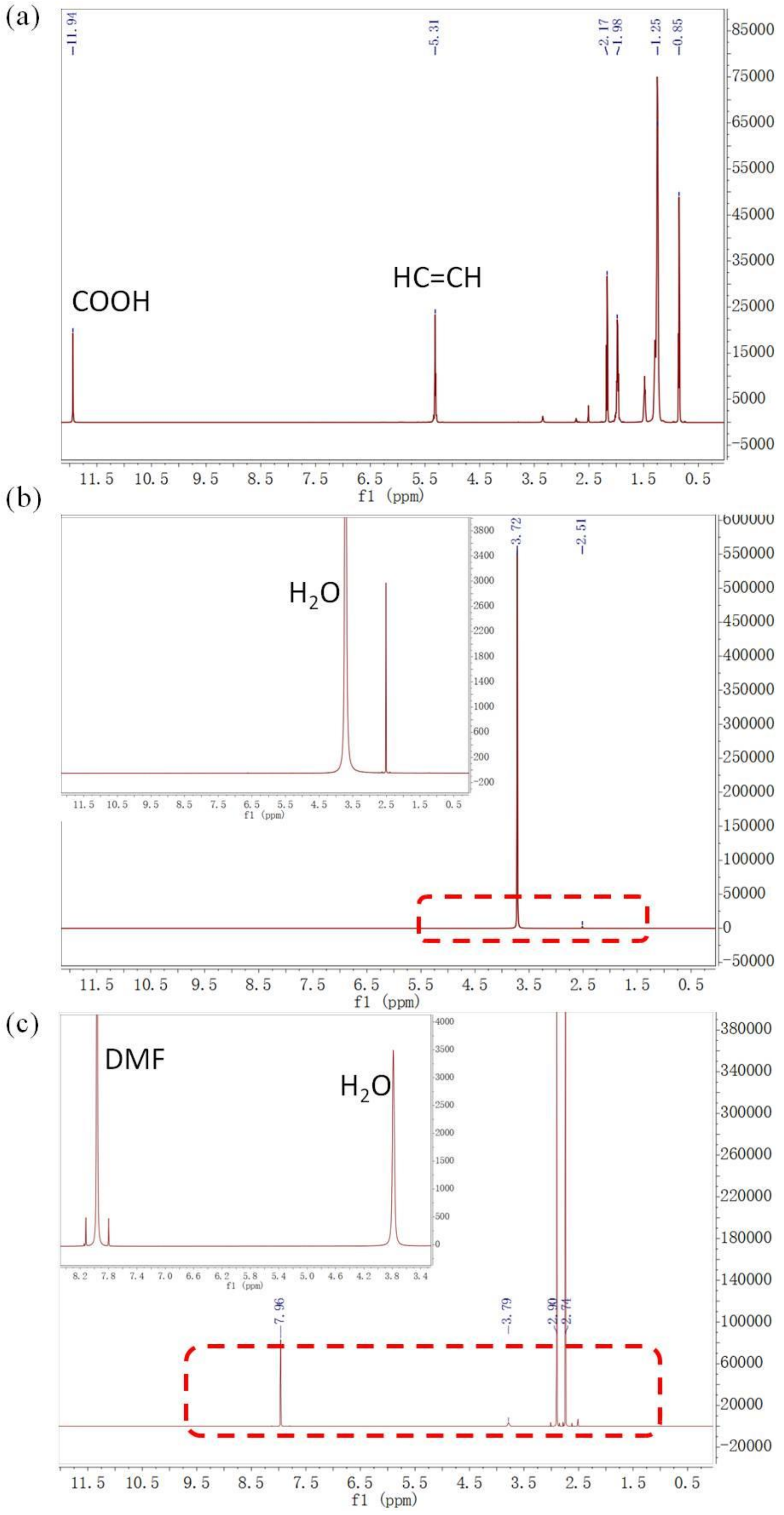

Fig. S3 ^{1}H NMR for **a** oleic acid, **b** Deionized H_2O and **c** 45% Hydroiodic acid mixed with DMF in d_6-DMSO. Spectra referenced to residual nondeuterated dimethyl sulfoxide (DMSO) peak at ~2.5 ppm. Peaks belonging to oleate acid are CH_3 (∂ = 0.85 ppm), CH_2 (∂ =1.44 – 2.71 ppm), HC = CH (∂ = 5.31 ppm) and COOH (∂ = 11.94 ppm). Peak belonging to Deionized H_2O is about 3.72 ppm. The Peaks at ∂ = 2.5-2.9 and 7.96 ppm are caused by DMF

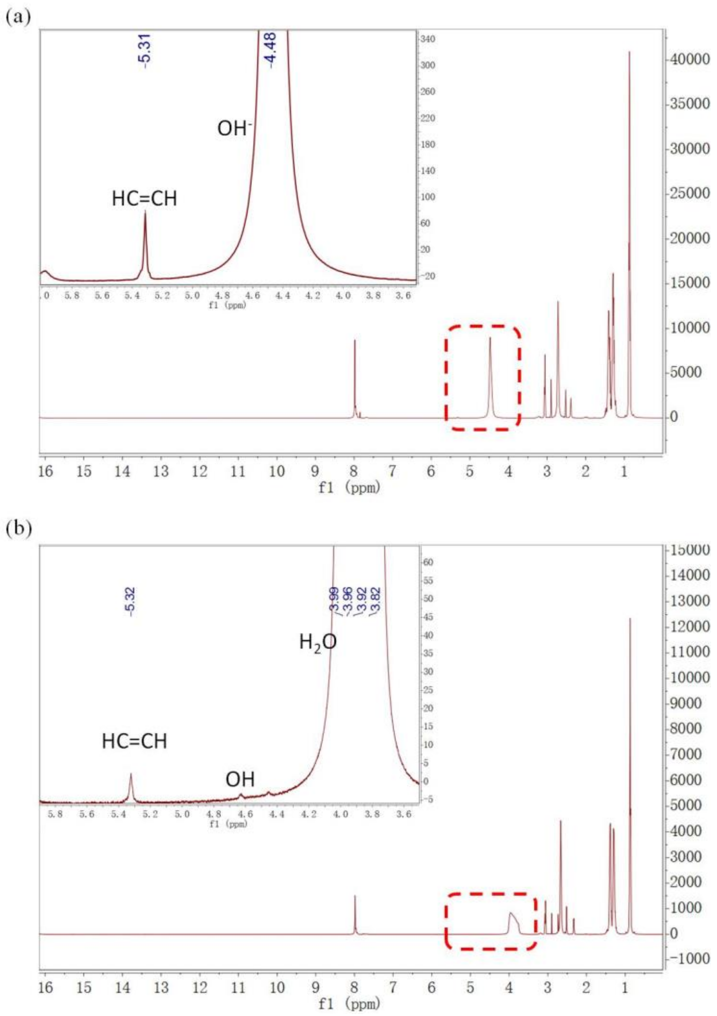

Fig. S4 ^{1}H NMR for **a** w/o HI-PbS and **b** w/ HI-PbS QDs dispersed in d_6-DMSO

The w/ and w/o PbS-QDs were firstly dispersed in BTA. After completely drying the QDs solution, the treated PbS-QDs powders were re-dissolved in d_6-DMSO and were filtered in glass test tube for measurement. Comparing with backgrounds NMR spectra (Fig. S3), the most notable changes were no free oleic acid peak at 11.94 ppm and significantly reduced oleate at 5.32 ppm for both w/ and w/o PbS-QDs (Fig. S4), indicating the original oleate or oleic acid was significantly removed. The peak for OH (∂ = 4.48 ppm) could be easily found in w/o HI-PbS but not clear in w/ HI-PbS, and then, the w/ QDs obtained a peak at 3.6-4.2 ppm is H signal from H_2O, which further prove the existence of H_2O from the deprotonation reaction between OH group and HI.

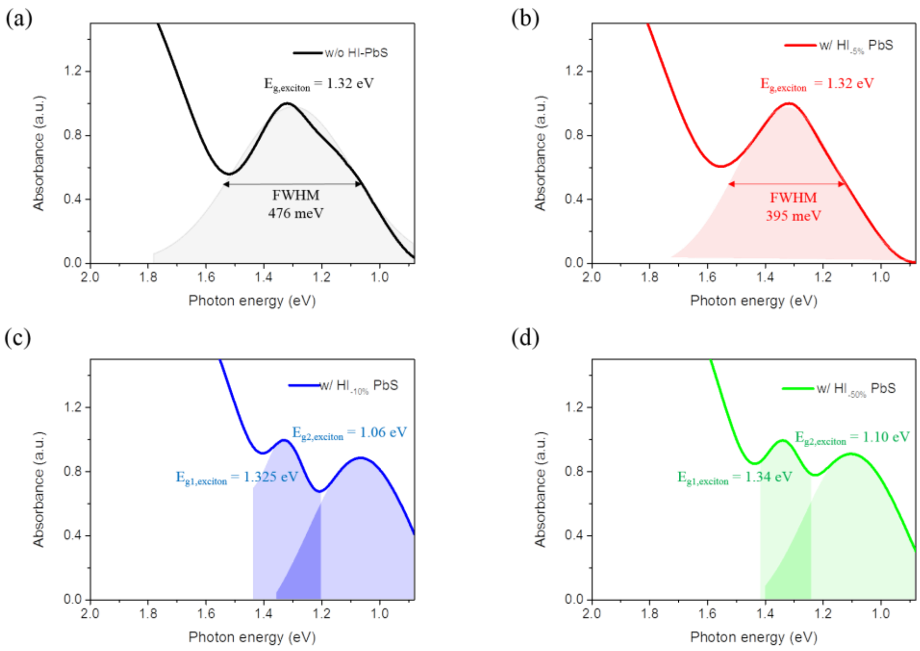

Fig. S5 Absorbance spectra of PbS-QDs films with different ratio of **a** 0%-HI, **b** 5%-HI, **c** 10%-HI and **d** 50%-HI treatment

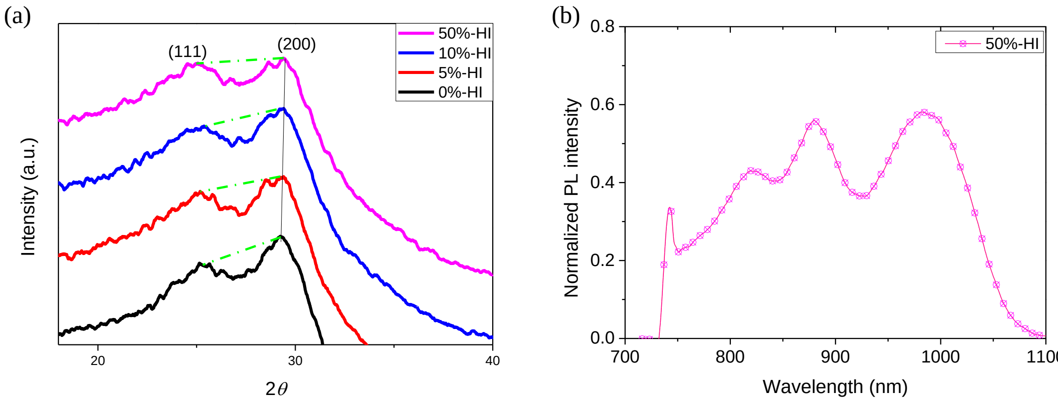

Fig. S6 a X-ray diffraction patterns of the PbS-QDs films with different ratio for HI treatment, indicating the changes of the treated PbS-QDs size. **b** Steady state PL spectrum of 50% HI adding in PbI_2-DMF ligand solution for treating PbS CQDssolution, the PL intensity was normalized to the absorption of this solution

As shown in XRD spectra, it should be noted that CQDs treated by HI solution with more ratio (50%) showed broader peak and similar intensity of (111) and (200) peak, which was generally observed in smaller QDs [S11, S12]. For PL measurement, there was a part of short wavelength PL appearing in w/ $HI_{-50\%}$ PbS-QDs solution, which was considered as an indication of decomposition in PbS-CQDs films (Fig. S6b). Therefore, it further illustrated more HI treatment would decompose the CQDs.

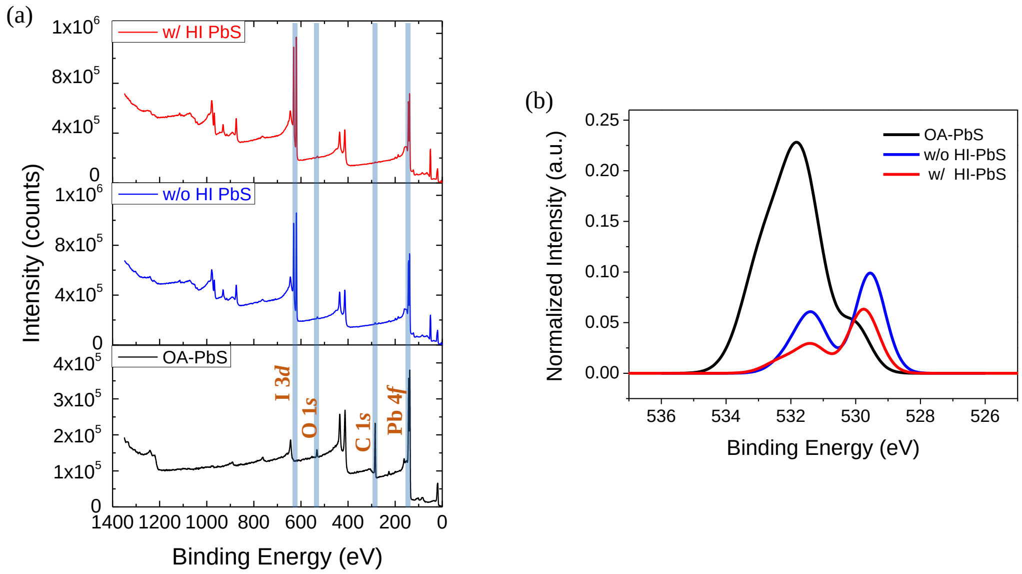

Fig. S7 a Full XPS spectra of different various PbS-QDs film. **b** O 1s XPS of OA-PbS, w/o and w/ HI PbS-QDs films. The peak intensities were normalized to the Pb peak area of the corresponding sample

Table S1 Atomic ratios of OA-PbS, w/o and w/ HI PbS-QDs films

Condition	Pb	S	O	I	C
**OA-PbS**	1	0.85	0.54	0	7.74
**w/o HI PbS**	1	0.68	0.21	0.65	0.33
**w/ HI PbS**	1	0.60	0.14	0.76	0.14

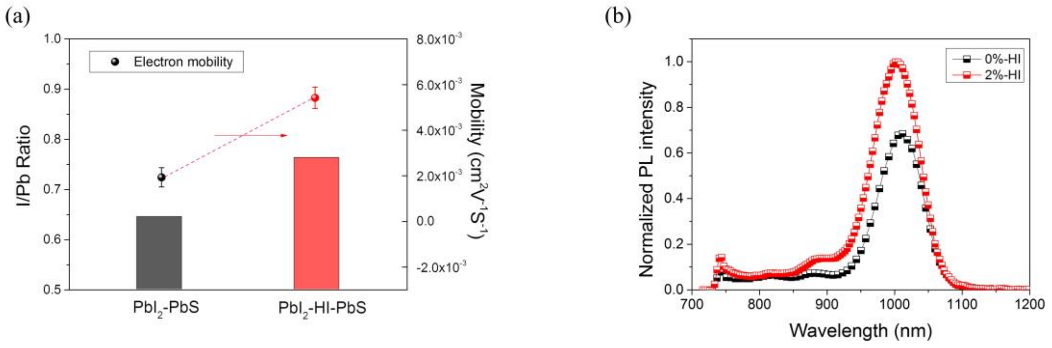

Fig. S8 a Composition analysis of w/o and w/ HI PbS-QDs films from the XPS results. **b** Steady state PL spectra of 0% and 2% HI adding in PbI_2-DMF ligand solution for treating PbS CQDs solutions, the intensity was normalized to the absorption of the solutions

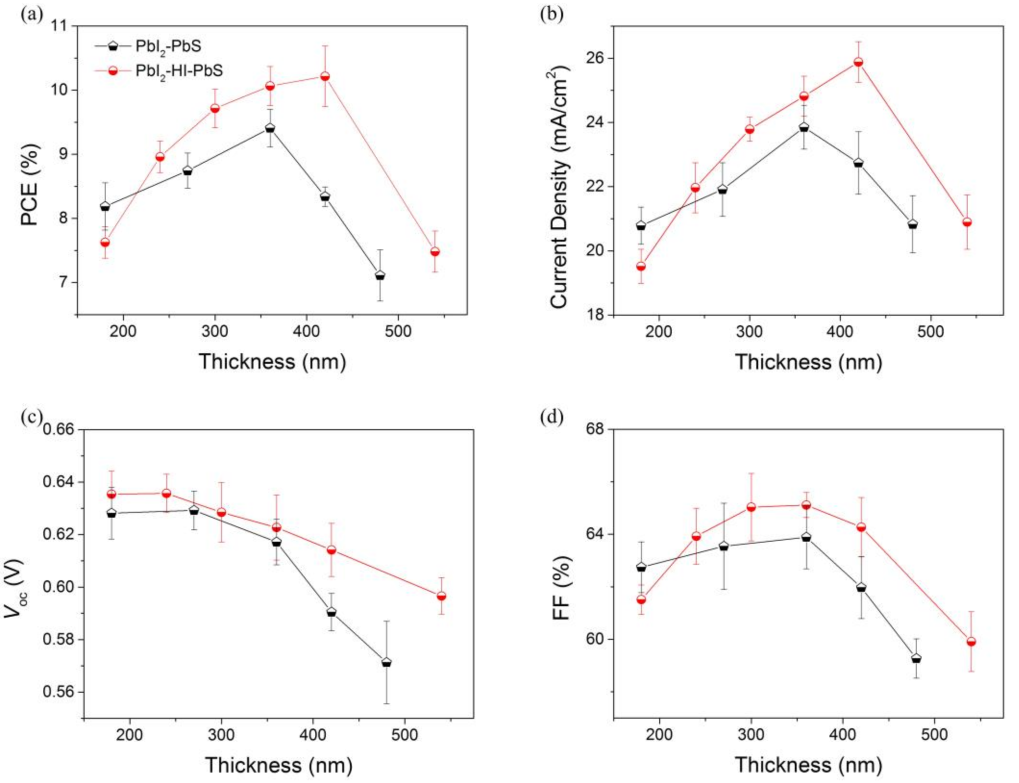

Fig. S9 Device performances with different active layer thickness based on w/o and w/ HI solution ligand exchange

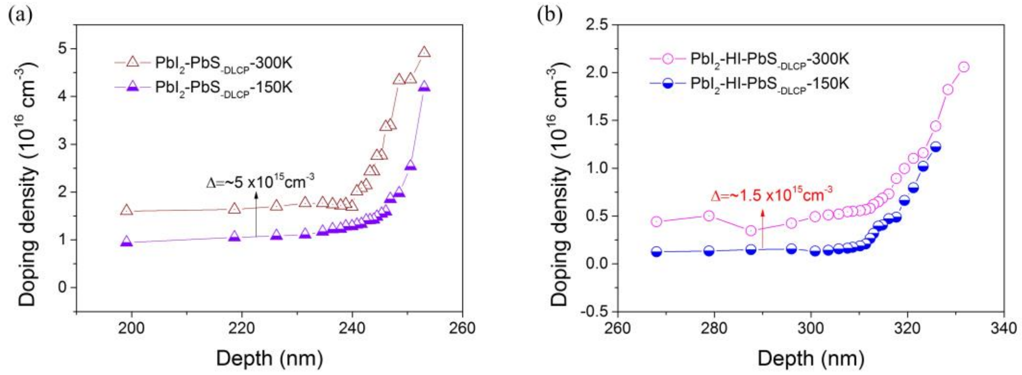

Fig. S10 DLCP profiling of 300 K and 150 K temperature for **a** w/o and **b** w/ HI PbS-QDs based devices

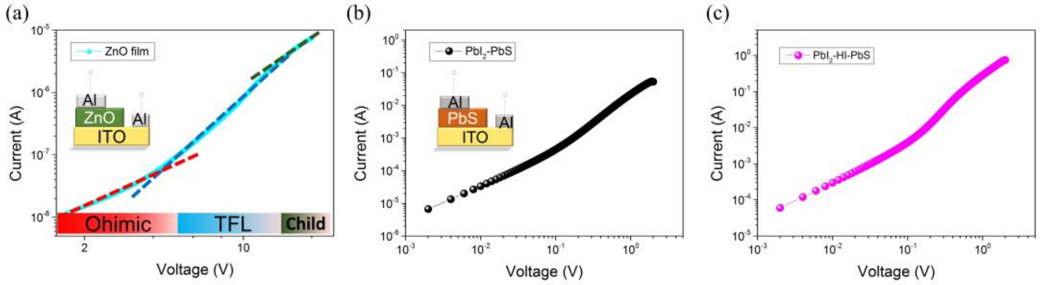

Fig. S11 SCLC electron only devices analysis result for **a** ZnO film**, b** w/o HI PbS-QDs and **c** w/ HI PbS-CQD devices

According to Eqs. S1 and S2, we can extract the electron mobility and bulk trap density of w/o and w/ HI PbS-QDs films, the μ_e are 1.9×10^{-3} and 5.27×10^{-3} cm^2 V^{-1} S^{-1}, the bulk trap density of both devices are $N_{w/o\ HI}$ = 1.1×10^{16} cm^3 and $N_{w/\ HI}$ = 4.1×10^{15} cm^3 respectively.

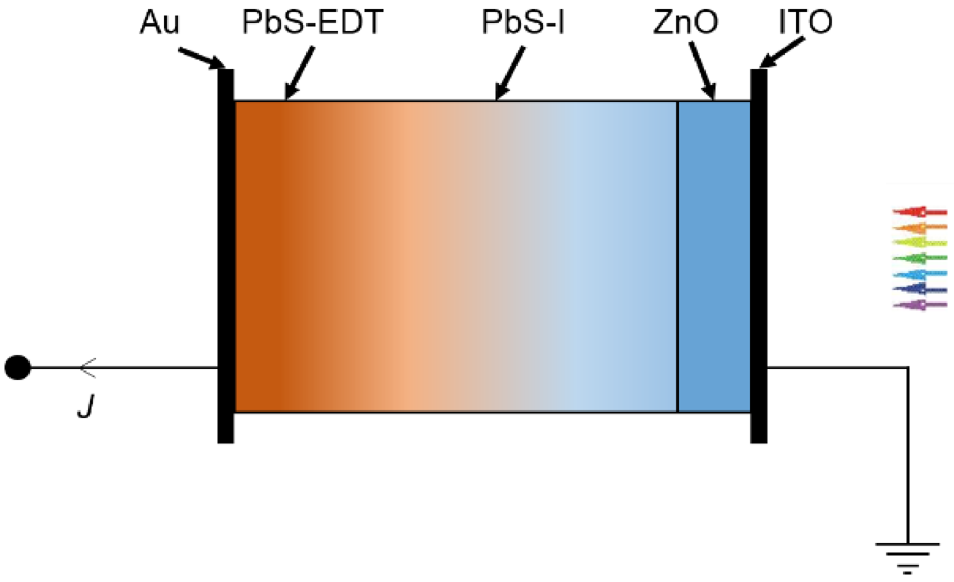

Fig. S12 SCAPS modelling parameters

Table S2 SCAPS modelling parameters

	PbS layer graded from PbS-EDT to PbS-I	ZnO
Thickness (nm)	350 nm or 420 nm	50
Bandgap (eV)	1.32 eV	3.25
Electron affinity (eV)	Graded from 3.9 to 4.15 (Parabolic Form)	4.2
Dielectric permittivity (relative)	20	66
CB effective density of states (1/cm^3)	1E19	1E19
VB effective density of states (1/cm^3)	1E19	1E19
Electron thermal velocity (cm/s)	7E3	1E7
Hole thermal velocity (cm/s)	7E3	1E7
Electron mobility (cm^2/V·S)	2E-2	5E-2
Hole mobility (cm^2/V·S)	2E-2	5E-2
Shallow uniform donor density N_D (1/cm^3)	Graded from 1E12 to 5E15 (Exponential form)	2E16
Shallow uniform donor density N_A (1/cm^3)	Graded from 1E16 to 1E12 (Exponential form)	1E14
Absorption coefficient (cm/s)	File from Figure S1	File
Radiative recombination coefficient (cm^3/s)	5E-13	0
Auger capture coefficient (cm^6/s)	1E-28	0
Trap capture cross section (cm^2)	1.2E-13	1E-14
Trap depth relative to Ec (eV)	0.5	0.3
Trap density (1/cm^3)	1E16 or 1E15	5E15

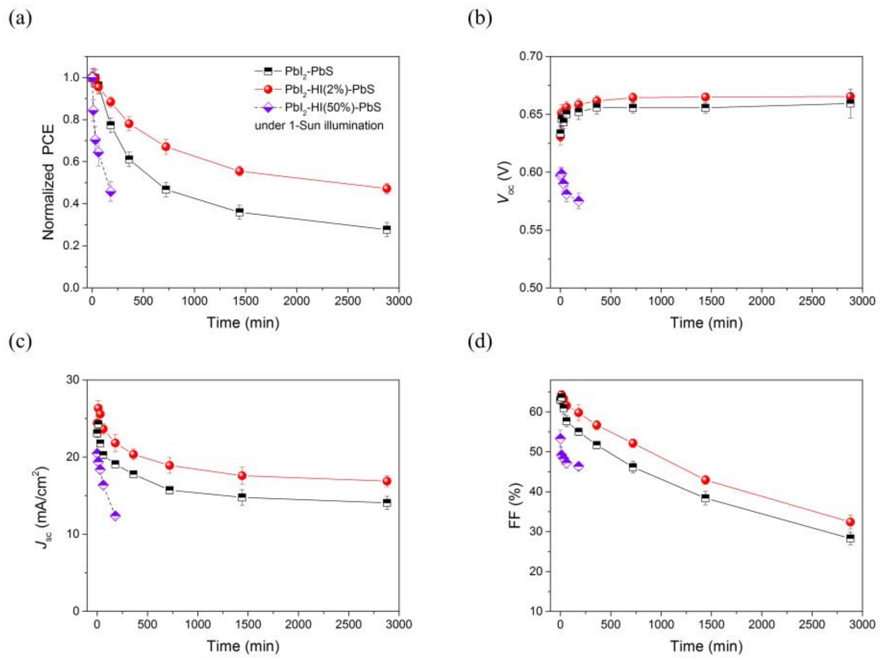

Fig. S13 Stability test of these devices under continuous AM 1.5G illumination. Storing time evolution without encapsulation of **a** normalized PCE, **b** V_{oc}, **c** J_{sc}, and **d** FF for w/o HI PbS and w/ HI PbS devices. The humidity and temperature were typically in the range of 30–50% and 27–30 °C

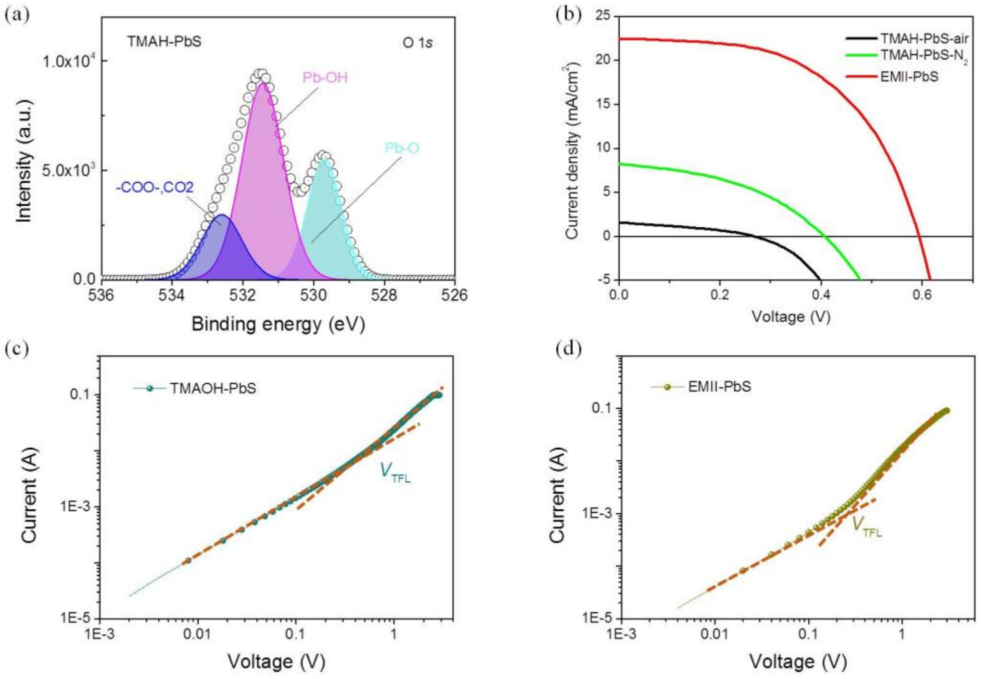

Fig. S14 a XPS spectra for deconvolution O 1*s* peak from TMAH treated PbS-QDs film. The real intensity of this peak was subtracted with background line signal. **b** *J*-*V* measurement for EMII and TMAH capped PbS-QDs solar cell under AM 1.5G illumination. Device performance for EMII and TMAH capped PbS-QDs with L-B-L method. SCLC measurements for **c** TMAH and **d** EMII treated PbS-QDs with hole and electron only device respectively.

The TMAH treated PbS-QDs were performed X-ray photoelectron spectroscopy (XPS) to investigate the species in QD films. The O 1*s* signal of XPS spectrum for EMII treated PbS-QDs film was referred to the previous work [S13], the Pb-OH peak was greatly suppressed and the COO^- peak was the highest signal for O 1*s* peak in EMII treated QDs film. By comparison, the highest signal for O 1*s* was from Pb-OH peak in TMAH treated PbS-QDs film, indicating TMAH treated PbS-QDs film had more OH ligands than EMII treated one [S13]. According to Eq. S1, we can extract the bulk trap density of TMAH and EMII treated PbS-QDs films, the bulk trap density of both devices are $N_{\mathrm{TMAH}} = 4.3\times10^{16}$ cm^3 and $N_{\mathrm{EMII}} = 2.1\times10^{16}$ cm^3, respectively.

Table S3 Performance of merit for the various treated PbS-QDs devices

**Device**	**PCE**	V_{oc}	J_{sc}	**FF**	N_{trap} **from DLCP**	N_{trap} **from SCLC**
**TMAH-air**	0.15	0.27	1.60	0.35	-	$\sim 4.3\times10^{16}$
**TMAH-N$_2$**	1.42	0.41	8.23	0.43		
**EMII**	7.24	0.59	22.44	0.55	-	$\sim 2.1\times10^{16}$
**w/o HI**	9.56%	0.62	24.48	0.63	$\sim 5\times10^{15}$	$\sim 1.1\times10^{16}$
**w/ HI**	10.78%	0.65	25.26	0.66	$\sim 1.5\times10^{15}$	$\sim 4.1\times10^{15}$

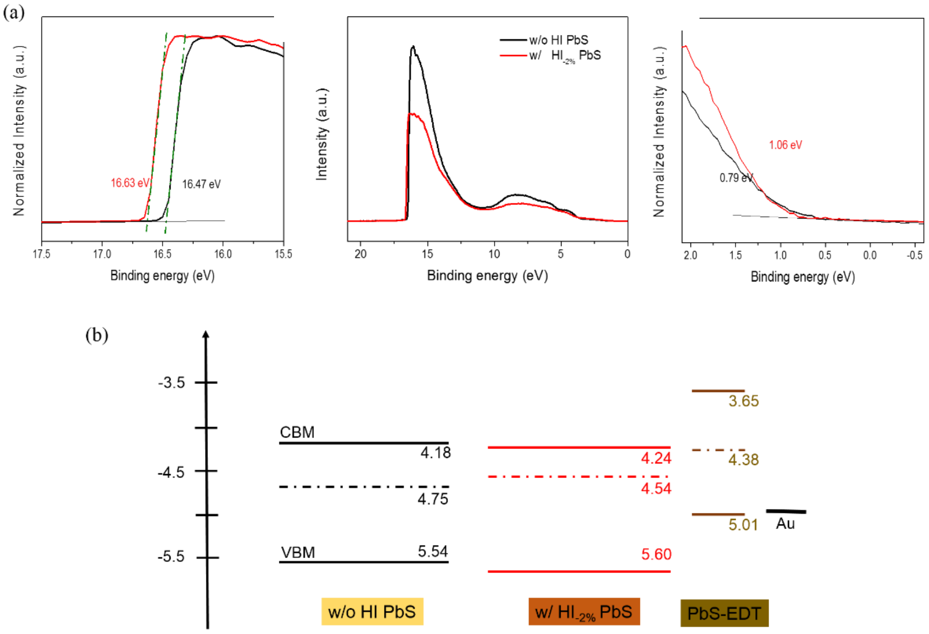

Fig. S15 a UPS of PbS QDs layer processed with w/o and w/ HI solution ligand exchange. The left panel shows the magnified view of the high binding energy region and the right panel shows the magnified view of the low binding energy region. Lines represent the linear fits. **b** The bottom figure shows the corresponding band position of w/o and w/ HI additive treated PbS-QDs films

From the XPS results, the I:Pb ratio had increased by w/ HI treatment. This change may affect the energy band alignment for device, we performed UPS measurements on w/o and w/ HI films to investigate it. As shown in Fig. S15a, the Fermi level of QDs film treated by HI shifted to shallower energy levels ascribed to higher iodine bonding content compared to w/o HI ones. The conduction band edge, Fermi level and valence band edge values for w/ HI film were -4.24, -4.54, and -5.60 eV, respectively. The energy level alignment at the PbS-QDs was plotted in Fig. S15b.

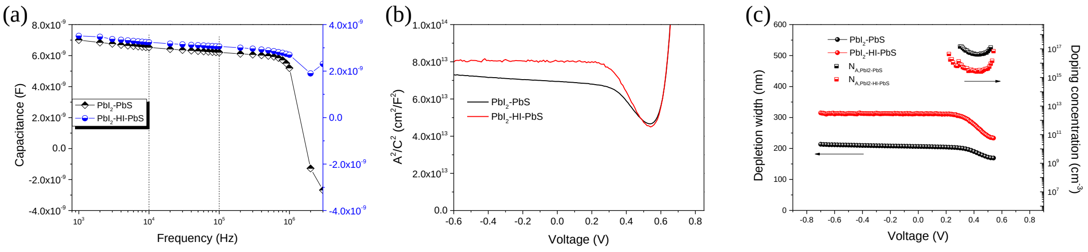

Fig. S16 a *C-f* measurement data of ITO/PbS/Al ranging from 10^3 Hz to 10^6 Hz. **b** The Mott–Schottky plots and **c** depletion results and $N_{\text{c-v}}$ of w/o and w/ HI PbS-CQDs

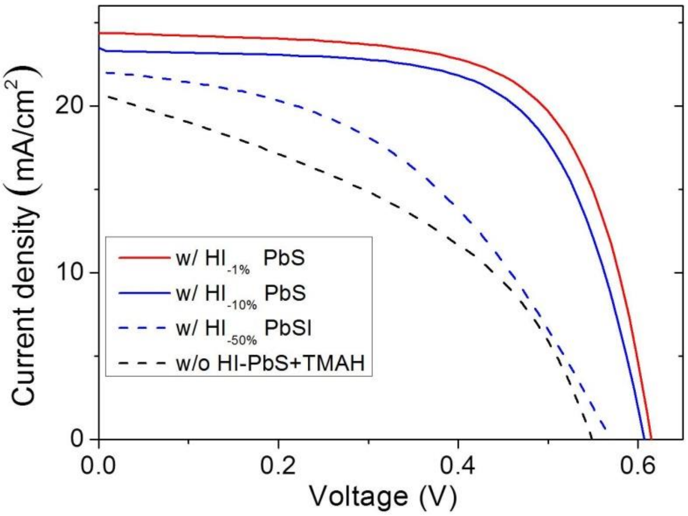

Fig. S17 *J-V* curves of solar cells based on PbS-QDs treated by various amount of HI acid or TMAH solution

Table S4 The related parameters of Figs. S17 and 5b with different mole ratio of HI acid based on w/ HI PbS-QDs solar cells under simulated AM 1.5 Gillumination conditions

Condition	J_{sc}	V_{oc}	FF (%)	PCE (%)
**0%-HI (w/o HI)**	24.48	0.62	0.63	9.56
**1%-HI**	24.67	0.615	0.66	10.01
**2%-HI (w/ HI)**	25.26	0.65	0.66	10.78
**10%-HI**	23.82	0.606	0.65	9.38
**50%-HI**	21.11	0.562	0.48	5.70
**w/o HI-PbS+TMAH**	20.72	0.549	0.42	4.72

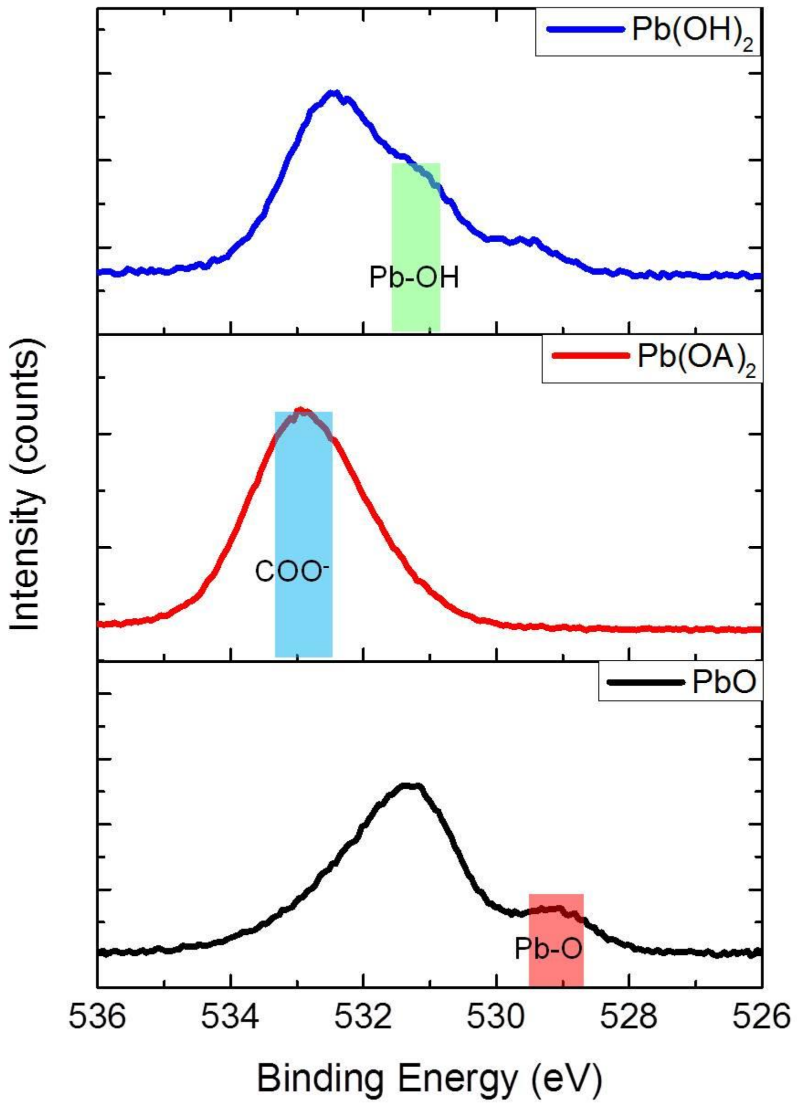

Fig. S18 The XPS spectra of three pure reference materials. O 1*s* XPS showing peaks at 529.1 eV from PbO, at 531 eV from O in Pb-OH and at 532.8 eV from O in carboxyl group. All of these peaks is broader caused by background, this broader peak for pure material is also found in previous work [S14]

Supplementary References

[S1] X. Yang, L. Hu, H. Deng, K. Qiao, C. Hu et al., Improving the performance of PbS quantum dot solar cells by optimizing ZnO window layer. Nano-Micro Lett. **9**(2), 24 (2017). http://doi.org/10.1007/s40820-016-0124-2

[S2] J. Tang, K.W. Kemp, S. Hoogland, K.S. Jeong, H. Liu et al., Colloidal-quantum-dot photovoltaics using atomic-ligand passivation. Nat. Mater. **10**(10), 765-771 (2011). http://doi.org/10.1038/nmat3118

[S3] H. Aqoma, M. Al Mubarok, W.T. Hadmojo, E.H. Lee, T.W. Kim et al., High-efficiency photovoltaic devices using trap-controlled quantum-dot ink prepared via phase-transfer exchange. Adv. Mater. **29**(19), 1605756 (2017). http://doi.org/10.1002/adma.201605756

[S4] Q. Dong, Y. Fang, Y. Shao, P. Mulligan, J. Qiu, C. Lei, J. Huang, Electron-hole diffusion lengths > 175 μm in solution-grown $CH_3NH_3PbI_3$ single crystals. Science **347**(6225), 967-970 (2015). http://doi.org/10.1126/science.aaa5760

[S5] Y. Liu, Z. Yang, D. Cui, X. Ren, J. Sun et al., Two-inch-sized perovskite $CH_3NH_3PbX_3$ (X = Cl, Br, I) crystals: growth and characterization. Adv. Mater. **27**(35), 5176-5183 (2015). http://doi.org/10.1002/adma.201502597

[S6] J. Khan, X. Yang, K. Qiao, H. Deng, J. Zhang et al., Low-temperature-processed SnO_2–Cl for efficient PbS quantum-dot solar cells via defect passivation. J. Mater. Chem. A **5**(33), 17240-17247 (2017). http://doi.org/10.1039/C7TA05366E

[S7] C. Wei, W. Yongzhen, Y. Youfeng, L. Jian, Z. Wenjun et al., Efficient and stable large-area perovskite solar cells with inorganic charge extraction layers. Science **350**(6263), 944-948 (2015). http://doi.org/10.1126/science.aad1015

[S8] J.T. Heath, J.D. Cohen, W.N. Shafarman, Bulk and metastable defects in $CuIn_{1-x}GaxSe_2$ thin films using drive-level capacitance profiling. J. Appl. Phys. **95**(3), 1000-1010 (2004). http://doi.org/10.1063/1.1633982

[S9] M. Burgelman, K. Decock, S. Khelifi, A. Abass, Advanced electrical simulation of thin film solar cells. Thin Solid Films **535**, 296-301 (2013). http://doi.org/10.1016/j.tsf.2012.10.032

[S10] X. Lan, O. Voznyy, A. Kiani, F.P. Garcia de Arquer, A.S. Abbas et al., Passivation using molecular halides increases quantum dot solar cell performance. Adv. Mater. **28**(2), 299–304 (2016). http://doi.org/10.1002/adma.201503657

[S11] J. Tang, L. Brzozowski, D.A.R. Barkhouse, X. Wang, R. Debnath et al., Quantum dot photovoltaics in the extreme quantum confinement regime: the surface-chemical origins of exceptional air- and light-stability. ACS Nano **4**(2), 869-878 (2010). http://doi.org/10.1021/nn901564q

[S12] Z. Ning, H. Dong, Q. Zhang, O. Voznyy, E.H. Sargent, Solar cells based on inks of n-type colloidal quantum dots. ACS Nano **8**(10), 10321-10327 (2014).

http://doi.org/10.1021/nn503569p

[S13] Y. Cao, A. Stavrinadis, T. Lasanta, D. So, G. Konstantatos, The role of surface passivation for efficient and photostable PbS quantum dot solar cells. Nat. Energy **1**(4), 16035 (2016). http://doi.org/10.1038/nenergy.2016.35

[S14] D. Zherebetskyy, M. Scheele, Y. Zhang, N. Bronstein, C. Thompson et al., Hydroxylation of the surface of PbS nanocrystals passivated with oleic acid. Science **344**(6190), 1380-1384 (2014). http://doi.org/10.1126/science.1252727
